# Conditioned Pain Modulation Inter‐Site Variability Study: Effect Sizes and Test–Retest Reliability of Two Models

**DOI:** 10.1002/ejp.70287

**Published:** 2026-05-04

**Authors:** Yi‐Wun Lin, Hannah Schmidt, Niko Möller‐Grell, Walter Magerl, Li‐Ling Hope Pan, Shuu‐Jiun Wang, Li‐Wei Chou, Rolf‐Detlef Treede

**Affiliations:** ^1^ Department of Neurophysiology Mannheim Center of Translational Neuroscience (MCTN), Ruprecht Karls University Heidelberg Mannheim Germany; ^2^ Department of Physical Therapy and Assistive Technology National Yang Ming Chiao Tung University Taipei Taiwan; ^3^ Department of Psychiatry and Psychotherapy, Central Institute of Mental Health (CIMH) Ruprecht Karls University Heidelberg Mannheim Germany; ^4^ Brain Research Center National Yang Ming Chiao Tung University Taipei Taiwan; ^5^ Department of Neurology, Neurological Institute Taipei Veterans General Hospital Taipei Taiwan; ^6^ School of Medicine, College of Medicine National Yang Ming Chiao Tung University Taipei Taiwan

**Keywords:** conditioned pain modulation, heat pain sensitivity, pressure pain threshold, reference data, test–retest reliability

## Abstract

**Background:**

Conditioned pain modulation (CPM) is a set of psychophysical paradigms that is increasingly used clinically to evaluate descending pain modulation pathways. Impairment is common in chronic pain, suggesting CPM may serve as a mechanistic indicator. However, the lack of protocol standardization and reference data prevents clinical use in individual patients.

**Methods:**

We compared two CPM protocols with different conditioning stimulus intensities, test stimulus types, and interaction timing. We assessed CPM effect size, test–retest reliability and sensitivity to detect loss of descending inhibition.

**Results:**

Conditioning with 0°C water led to stronger inhibition of pressure pain threshold (PPT) than conditioning with 7°C water (Cohen's d = 0.52), when tested immediately after conditioning. When tested during conditioning, effects of 7°C water immersion on heat pain sensitivity had similar magnitude (*D* = 0.53) and test–retest reliability (ICC = 0.77) as those on PPT (*D* = 0.54, ICC = 0.73). For all outcomes assessed, 95% confidence intervals (CI) of CPM effect included some facilitation instead of inhibition. The maximum degree of facilitation compatible with normal CPM (upper cutoff of CI) indicates potential sensitivity to detect individual abnormality. This was most favourable for PPT assessed after conditioning with 0°C water (decrease by more than 75 kPa or 14% of baseline PPT).

**Conclusions:**

In conclusion, testing during conditioning stimulation yields medium to large effect sizes and good test–retest reliability. PPT testing immediately after ice water immersion has the narrowest 95% CI and hence offers the potential to generalize CPM assessments beyond group‐level differences and compare inhibition among individuals in clinical practice.

**Significance Statement:**

Indicating the main aspects where this work adds significantly to existing knowledge in the field, and if appropriate to clinical practice. Simultaneous CPM protocols exhibit large effect sizes but are confounded by divided attention. We recommend a sequential protocol and provide model reference data for abnormal facilitation.

**Abbreviations**
BMI: body mass index
CI: confidence interval
CPM: conditioned pain modulation
CSI: Central Sensitization Inventory
*D*
: Cohen's *d*
DFNS: Deutscher Forschungsverbund Neuropathischer Schmerz
DH: dominant hand
DNIC: diffuse noxious inhibitory control
HADS: Hospital Anxiety and Depression Scale
HPS: heat pain sensitivity
ICC: intra‐class correlation
IPAQ: International Physical Activity Questionnaire
kPa: kilopascals
LOC: Local Test Protocol
NDH: non‐dominant hand
NRS: Numerical Rating Scale
PCS: Pain Catastrophizing Scale
PPT: pressure pain threshold
PSQ: Pain Sensitivity Questionnaire
PSQI: Pittsburgh Sleep Quality Index
QST: quantitative sensory testing
SD: standard deviation
STD: standard test protocol

## 1 Introduction

Conditioned pain modulation (CPM) is a psychophysical paradigm used to evaluate descending pain modulation pathway. CPM is thought to activate spino‐bulbo‐spinal tracts, engaging mechanisms known as diffuse noxious inhibitory control (DNIC) in rodents (Ramaswamy and Wodehouse 2021; Sirucek et al. 2023). In both DNIC and CPM protocols, a noxious conditioning stimulus inhibits processing of other painful stimuli elsewhere in the body, reflecting descending pathways often impaired in neuropathic and chronic pain conditions (Staud 2012). Thus, CPM may serve as a mechanistic indicator of pain processing, a predictor of pain chronicity, and a method to monitor therapeutic progress (Granovsky 2013; Lewis et al. 2012; Schuh‐Hofer, Eichhorn, et al. 2018). Despite strong theoretical basis and demonstrated impairments in various chronic pain conditions, the clinical adoption of CPM remains limited due to methodological inconsistencies and variable reliability. Although studies have shown reduced CPM efficacy in chronic pain, the magnitude of this effect and its correlation with clinical symptoms remain inconsistent (Fernandes et al. 2019). Nevertheless, CPM continues to hold promise as a mechanistic biomarker provided that future studies address these implementation barriers through methodological refinement and normative data development (Demetriou et al. 2025; Nuwailati et al. 2022). Consequently, there is a strong interest in using CPM in clinical testing.

Studies on CPM use a wide range of different protocols that vary with respect to type and intensity of conditioning stimulus, type of test stimulus, and timing of evaluating their interactions (Ramaswamy and Wodehouse 2021). The choice of test and conditioning stimuli, as well as the intensity of conditioning pain, significantly influence CPM effect size (Granot et al. 2008; Ibancos‐Losada et al. 2020; Nuwailati et al. 2022). Some studies suggest that conditioning cold pain and pressure pain as test stimuli generate highly sensitive CPM results (Ibancos‐Losada et al. 2020). However, there is no widely accepted protocol for CPM assessment. To facilitate comparisons across studies from different laboratories, an expert panel has proposed a reporting standard (Yarnitsky et al. 2015).

CPM is a dynamic variant of Quantitative Sensory Testing (QST). For static QST, the German Research Network on Neuropathic Pain (DFNS) had agreed on a standard protocol and provided multicentre reference data and an algorithm to determine an abnormal finding in patients (Pfau et al. 2014). These steps were essential to move QST from a scientific tool to widespread clinical use. The lack of standardization and reference data has prevented the introduction of CPM testing into routine clinical practice.

This study was performed as part of consortium ‘CPM inter‐site variability study’, registered as CCMO‐NL74710.058.20. The primary goal of this study is to assess whether a Local Test Protocol (LOC) of participating laboratory (Eichhorn et al. 2018; Schuh‐Hofer, Eichhorn, et al. 2018) gives the same testing validity as a standard test protocol (STD) common to all studies (Ablin et al. 2023; Argaman et al. 2020; Granovsky et al. 2022). We quantify the factors contributing to variability in CPM and examine the test–retest reliability. In addition, we aim to provide proof‐of‐concept of how to derive clinical cutoffs for abnormal findings. A minimum inhibition cannot be set, since healthy subjects also show facilitation in CPM paradigms (Schliessbach et al. 2019; Vincenot et al. 2025). We thus evaluate the maximum amount of facilitation by calculating 95% confidence intervals of the range of normal variability in these CPM protocols as reference data from healthy participants. This analysis is intended to provide a model for other studies to report such confidence intervals as a basis for generalizing CPM assessments beyond group‐level differences and comparing inhibition among individuals in clinical practice.

## 2 Methods

### 2.1 Recruitment

Participants were recruited through posters, social media, and email invitations. The sample size was determined according to recommendations for single‐centre reference data, derived from the multi‐centre reference dataset of DFNS quantitative sensory testing protocol (Magerl et al. 2010). Our planned sample size of *n* = 20 is able to detect effect sizes above *D* = 0.66 (power = 0.8) for *t*‐tests in a crossover study. In the original DFNS reference dataset, participants were stratified into two age groups (≤ 40 years and > 40 years). Accordingly, the present study recruited healthy volunteers aged 18–40 years without clinical pain conditions. Mild and occasional headaches or other transient pain complaints were permitted if they had not persisted for longer than 3 months. Individuals who continuously use medications, as well as those who are pregnant or breastfeeding, were excluded. All tests were conducted in dedicated examination rooms within the human subject laboratories of the Department of Neurophysiology, maintained at a pleasant temperature, and noise‐shielded. Participants were unable to view any screens displaying performance. Prior to examination, a native German speaker provided detailed instructions regarding experimental procedures, expected sensations, and potential side effects, all of which had been disclosed in the informed consent form. The study had been approved by the local ethics committee (2014‐603N‐MA).

**FIGURE 1:**
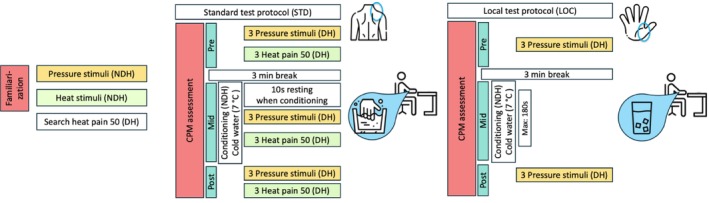
Study design for comparing two CPM protocols. Timeline for group A, visit 1. CPM, conditioned pain modulation; DH, dominant hand; Heat pain 50, heat stimulus that yields a rating of 50/100; HPS, heat pain sensitivity (suprathreshold rating); LOC, local CPM test protocol (ice water conditioning. CPM assessed by PPT after conditioning); NDH, non‐dominant hand; PPT, pressure pain threshold; STD, standard CPM test protocol (7°C water conditioning. CPM assessed by HPS and PPT during and after conditioning).

This is a two‐period cross‐over study, where all participants underwent two CPM protocols within each visit and the order was counterbalanced across the two visits. The sequence of protocols was randomized into two groups by drawing straws from a sealed, opaque container. Group A started the first visit with the standard comparator protocol (STD), and Group B began the first visit with the local protocol of our laboratory (LOC), Figure 1. Demographic, environmental, and psychological data were collected at the beginning of the first visit, followed by the two CPM protocols that were separated by a 30 min break. After completing the first visit, a minimum interval of 1 week was observed before performing the second visit with the two CPM protocols in reversed order to estimate the test–retest reliability (Geber et al. 2011, 2013).

The study was performed by two examiners (NMG, YWL), with the same examiner assigned to each subject throughout the study. The examiner read the instructions for both protocols from a standardized text. Before all tests, participants rated any pain in either upper limb using a 0–100 Numerical Rating Scale (NRS). This assessment accounts for any residual pain from a previous protocol. If pain more than NRS = 0 was reported, the next protocol was delayed until the residual pain subsided. The Numerical Rating Scale (NRS) scores perceived pain from zero to one hundred. Zero represents ‘no pain sensation’, and one hundred represents ‘the most imaginable pain’. Participants were allowed to use decimals in scoring.

### 2.2 Standard Test Protocol (STD)

There is no universally accepted standard protocol for CPM assessment. The ‘standard’ protocol in our study (STD) refers to an agreement among members of the informal consortium, which serves as a practical benchmark and anchor for comparisons across various local variants. In STD, CPM was tested on both upper limbs, utilizing two test stimuli on the dominant arm—thermal (volar forearm) and mechanical (trapezius muscle) and immersion of the non‐dominant hand in 7°C cold water as the conditioning stimulus (Granovsky et al. 2022; Lie et al. 2019; Nir and Yarnitsky 2015).

Initially, participants were familiarized with the Pressure Pain Threshold (PPT) and Heat Pain Sensitivity (HPS) tests. Participants were seated in a firm‐backed chair, and their blood pressure and pulse were measured before testing. The PPT test was performed with a 1 cm diameter probe (Wagner Instruments Inc., Greenwich, CT). Pressure stimuli were applied to the dominant trapezius at the neck‐shoulder junction, with a pressure increase rate of 0.5 kg/s (50 kPa/s); 1 s timing was provided by a metronome. Participants were instructed to verbally indicate when pressure pain onset occurred, at which point the stimulation was stopped.

For the HPS test, heat stimuli were applied to the non‐dominant volar mid‐forearm using a Thermal Sensory Analyser‐II with a 30 × 30 mm contact probe (Medoc Ltd. Advanced Medical Systems, Ramat Yishai, Israel). The maximum temperature range was up to 50°C. The testing sequence included a baseline temperature of 32°C, with a stimulus increase rate of 2°C/s and a decrease rate of 8°C/s. For familiarization, the temperature was raised in three steps: 43°C, 45°C, and 47°C, each maintaining a plateau for 7 s with an inter‐stimulus interval of 10 s. Participants were asked to rate the pain magnitude to ensure their understanding of the testing.

Following familiarization with the heat stimuli, the next step involved determining the Heat Stimulation Temperature that evokes perceived pain at a level of 50 on the 0–100 NRS (Pain50). This began with stimulation of the dominant forearm at temperatures between 45°C and 50°C, each for 7 s with an Inter‐stimulus Interval of 10 s. Each temperature was applied three times. The probe was slightly moved between stimuli, and pain ratings were obtained to determine the temperature that elicited a perceived pain rating of 50 on the 0–100 NRS (Pain50).

Data collection began before conditioning, using three pressure‐algometer stimuli administered to determine PPT at the dominant trapezius muscle. The probe was slightly moved between stimuli and allowed a 5‐s interval between stimuli. After this, HPS was assessed by administering a 20‐s heat stimulus at the Pain50 level determined before. HPS ratings were obtained at three specific time points: once the plateau temperature is reached (1st), at 8 s (2nd), and at 18 s (3rd).

The next phase of the study involved the application of the conditioning stimulus in conjunction with test stimuli. Participants immersed their non‐dominant hand in water maintained at 7°C. Ten seconds after initiating this conditioning stimulus, the previously described test stimuli—pressure followed by heat—were administered again. After applying these stimuli, the pain rating for the cold water immersion was obtained again and then the participant withdrew their hand. Immediately after withdrawal, the test stimuli were repeated.

### 2.3 Local Test Protocol (LOC)

In developing the LOC protocol, we aimed to establish a clinically relevant and methodologically robust model. We selected Pressure Pain Threshold (PPT) at the thenar eminence as the test stimulus due to its high test–retest reliability (*r* = 0.881), low inter‐individual variability, and ease of administration with simple and inexpensive tools (Rolke et al. 2005; Rolke, Baron, et al. 2006). Cold water immersion was chosen as the conditioning stimulus based on its frequent use in the CPM literature (Granovsky et al. 2022; Lie et al. 2019; Nir and Yarnitsky 2015); ice water at 0°C was used because of its frequent use for another purpose (cold pressor test) and its ease in handling technically. This pairing was designed to optimize both feasibility (no need for expensive equipment) and sensitivity for detecting changes in descending pain inhibition (see also meta‐analysis by Nuwailati et al. 2022). The sequential protocol was designed to minimize attentional distraction that may influence pain perception (Plaghki et al. 1994; Reinert et al. 2000; Sprenger et al. 2012; Torta et al. 2019). Sensitivity in eliciting CPM effects in clinical populations and sensitivity to change by chronic pain and its treatment was previously demonstrated for LOC in studies involving spinal cord stimulation and sleep deprivation (Eichhorn et al. 2018; Schuh‐Hofer, Eichhorn, et al. 2018; Schuh‐Hofer, Fischer, et al. 2018).

LOC involves several steps to ensure proper familiarization and accurate measurement of pain thresholds. Initially, to familiarize participants with the test, the PPT is applied to the thenar muscle of the dominant hand, differing from the trapezius muscle in STD. After a 5‐min interval, a PPT test is administered to establish the baseline measurement.

Following the baseline PPT assessment, participants immersed their non‐dominant hand in ice water maintained at 0°C. They were instructed to provide a Numerical Rating Scale (NRS) rating every 10 s until they reached a pain intensity rating of 100, at which point they withdrew their hand. Insensitive subjects are instructed to withdraw their hand at 180 s (Martel et al. 2024). The PPT is measured immediately upon withdrawing their hand from the ice water.

### 2.4 Participant Phenotyping by Psychosocial Variables

The study employed six questionnaires that assess various psychosocial factors previously found to influence pain perception and modulation. The Hospital Anxiety and Depression Scale (HADS) was used to measure levels of anxiety and depression among participants. Physical activity levels were evaluated using the short form of the International Physical Activity Questionnaire (IPAQ), which assesses average physical activity over the past 7 days. The Pittsburgh Sleep Quality Index (PSQI) measures sleep quality, an important factor associated with increased pain sensitivity and decreased pain tolerance. The Pain Catastrophizing Scale (PCS) investigated the likelihood of participants experiencing an exaggerated negative mindset towards pain, assessing the emotional impact of pain. The Pain Sensitivity Questionnaire (PSQ) measured sensitivity to pain in different imagined situations, providing pain sensitivity scores. The Central Sensitization Inventory (CSI) was used as a symptom‐based screening instrument to identify hypersensitivity features for pain and other percepts attributed to central nervous system sensitization.

All questionnaires were written in German; a native German speaker was available to answer participant queries. These six questionnaires were completed before the CPM test started.

### 2.5 Data Reporting and Analysis

An Excel template was used for standard data filing. Repetitions of the test stimuli (PPT, HPS) were averaged to obtain mean scores; the averaged three testing scores were used to calculate the average of a single test. CPM effects were calculated as differences from baseline such that negative values indicate inhibition (Yarnitsky et al. 2015). For HPS ratings, this was mid‐pre or post‐pre; for PPT thresholds, this was pre‐mid or pre‐post.

Intention‐to‐treat analysis was applied to all participants. Statistical analyses were performed by R Statistical Software (RStudio 2020). The Shapiro–Wilk test was used to assess the normality of distributions. *T*‐tests (Mann–Whitney tests for non‐normally distributed data) were conducted for continuous variables. Pearson's correlation was utilized to examine the relationships between variables.

For test–retest reliability, intra‐class correlation (ICC) estimates are based on a mean‐rating (*k* = 3), absolute‐agreement, 2‐way mixed‐effects model. ICC values are interpreted as follows: less than 0.5 indicates poor reliability, 0.5–0.75 indicates moderate reliability, 0.75–0.9 indicates good reliability, and greater than 0.9 indicates excellent reliability. We use Cohen's *d* as a measure of effect size, where 0.2 indicates a small effect, 0.5 is a medium effect, and 0.8 is a large effect (Cohen 2016). Statistical significance was established at *p* < 0.05.

As a first approximation to characterize CPM responses, participants were classified as either ‘responders’ or ‘non‐responders’ based on the direction of the CPM effect. Participants exhibiting an increase in PPT after conditioning were labelled as ‘CPM responders’, indicating effective engagement of endogenous analgesic mechanisms. In contrast, individuals with a decrease in PPT were labelled as ‘CPM non‐responders’, reflecting pain facilitation rather than inhibition. Other authors have suggested distinguishing three possible outcomes: inhibition, non‐response, and facilitation. In a large study using ice water conditioning effects in healthy subjects, an increase or decrease of forearm PPT by more than 5.3% has been proposed to indicate reliable inhibition or facilitation (Locke et al. 2014). However, we discourage the use of such terminologies, as they do not identify individual patient outcomes as being inside or outside the range of variability in healthy subjects.

Instead, 95% confidence intervals of CPM effects were calculated as mean ± 1.96 standard deviation (SD) as an estimate for reference data. Since inhibition is represented by CPM effects with negative signs (Yarnitsky et al. 2015), we were looking for an upper cutoff beyond which the lack of inhibition would be considered abnormal. Although group mean CPM effects in healthy subjects usually are negative (inhibitory), it is known that some subjects exhibit no inhibition or even some degree of facilitation (Locke et al. 2014; Vincenot et al. 2025). Hence the upper cutoff will indicate the maximum degree of facilitation compatible with having normal descending controls. This raises the question if there are limits to how much facilitation can result numerically from the two CPM protocols (theoretically possible ranges). HPS rating might change between the full range of 0–100 in both directions, PPT thresholds might change between the full range of 0–980.7 kPa in both directions. More realistically, expected changes may move from the mean pre‐values to these extreme values.

## 3 Results

**FIGURE 2:**
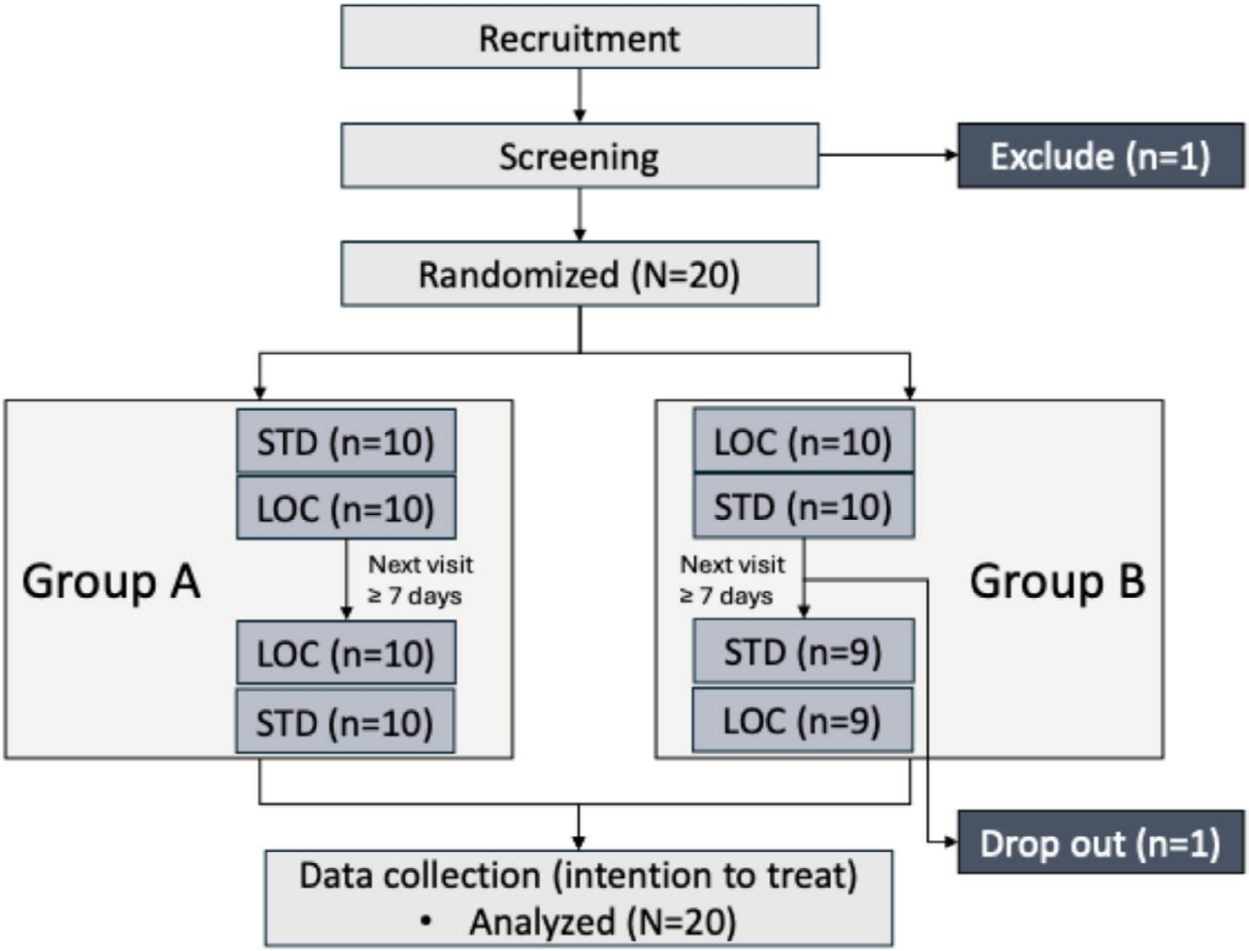
Participant disposition. This was a cross‐over study, where group A underwent STD before LOC in visit 1 and LOC before STD in visit 2. Group B underwent the inverse order for a balanced design. CPM, conditioned pain modulation; LOC, local CPM test protocol (ice water conditioning, CPM assessed by PPT after conditioning); STD, standard CPM test protocol (7°C water conditioning, CPM assessed by HPS and PPT during and after conditioning).

Data collection was conducted from August 4, 2022 to December 20, 2022. Twenty healthy subjects have been included in the study (10 each in group A and B, 7 females and 13 males). One subject dropped out after the first visit due to unwillingness to join in the repetition of the tests, leaving 39 assessments of CPM in total for each of the two protocols (Figure 2). All subjects were young adults (average age 23.3 y/o) with normal BMI (average 23.9 kg/m^2^). The room temperature was in the thermally neutral range (average 23.3°C). The questionnaire results found no significant group difference in demographic data (Table 1).

**TABLE 1:** Demographic data of the participants.

	All (*n* = 20)	All (*n* = 20)	Group A (*n* = 10)	Group B (*n* = 10)	*p*
		Female/Male [Cohen's *d*]			
Age (years)	23.3 ± 2.3	23.3 ± 1.8/23.1 ± 3.0 [0.06]	24.1 ± 2.4	22.5 ± 2.0	0.14
Sex (F/M)	7/13	—	3/7	4/6	0.66
Handedness (left–right)	3/17	—	3/7	0/10	0.21
Height (cm)	178 ± 11	—	178 ± 12	179 ± 9	0.95
Weight (kg)	76.4 ± 13.3	—	75.4 ± 12.7	77.4 ± 13.9	0.75
BMI (kg/m^2^)	23.9 ± 2.6	24.5 ± 2.6/24.0 ± 2.7 [0.20]	23.6 ± 1.6	24.2 ± 3.2	0.59
CSI	16.5 ± 7.1^a^	15.5 ± 6.8/17.2 ± 8.3 [0.21]^a^	18.7 ± 7.0^a^	14.6 ± 6.7	0.23
HADS (A)	3.5 ± 2.3^a^	2.9 ± 1.6/4.0 ± 2.8 [0.43]^a^	3.8 ± 2.4^a^	3.2 ± 2.0	0.56
HADS (D)	2.7 ± 2.2^a^	2.2 ± 1.3/3.1 ± 2.8 [0.40]^a^	2.8 ± 2.4^a^	2.6 ± 2.0	0.88
IPAQ (High/Moderate/Low)	16/3/0^a^	—	8/1/0^a^	10/0/0	0.60
PCS	5.4 ± 3.6^a^	6.7 ± 2.9/4.7 ± 4.3 [0.54]^a^	6.0 ± 3.5^a^	4.9 ± 3.7	0.54
PSQI	6.3 ± 2.0^a^	5.5 ± 1.7/6.7 ± 2.3 [0.59]^a^	6.3 ± 2.1^a^	6.3 ± 1.9	0.98
PSQ	3.1 ± 0.9^a^	3.3 ± 1.1/3.0 ± 0.9 [0.33]^a^	3.4 ± 1.0^a^	2.9 ± 0.7	0.20

*Note:* Results are presented as mean ± SD. *p*‐values from *t*‐tests and chi‐square tests.

Abbreviations: A, anxiety; BMI, body mass index; CSI, Central Sensitization Inventory; D, depression; HADS, Hospital Anxiety and Depression Scale; IPAQ, International Physical Activity Questionnaire; PCS, Pain Catastrophizing Scale; PSQI, Pittsburgh Sleep Quality Index PSQ, Pain Sensitivity Questionnaire.

^a^ Represents one data point missing.

### 3.1 Perceived Intensity of the Two Conditioning Stimuli

**FIGURE 3:**
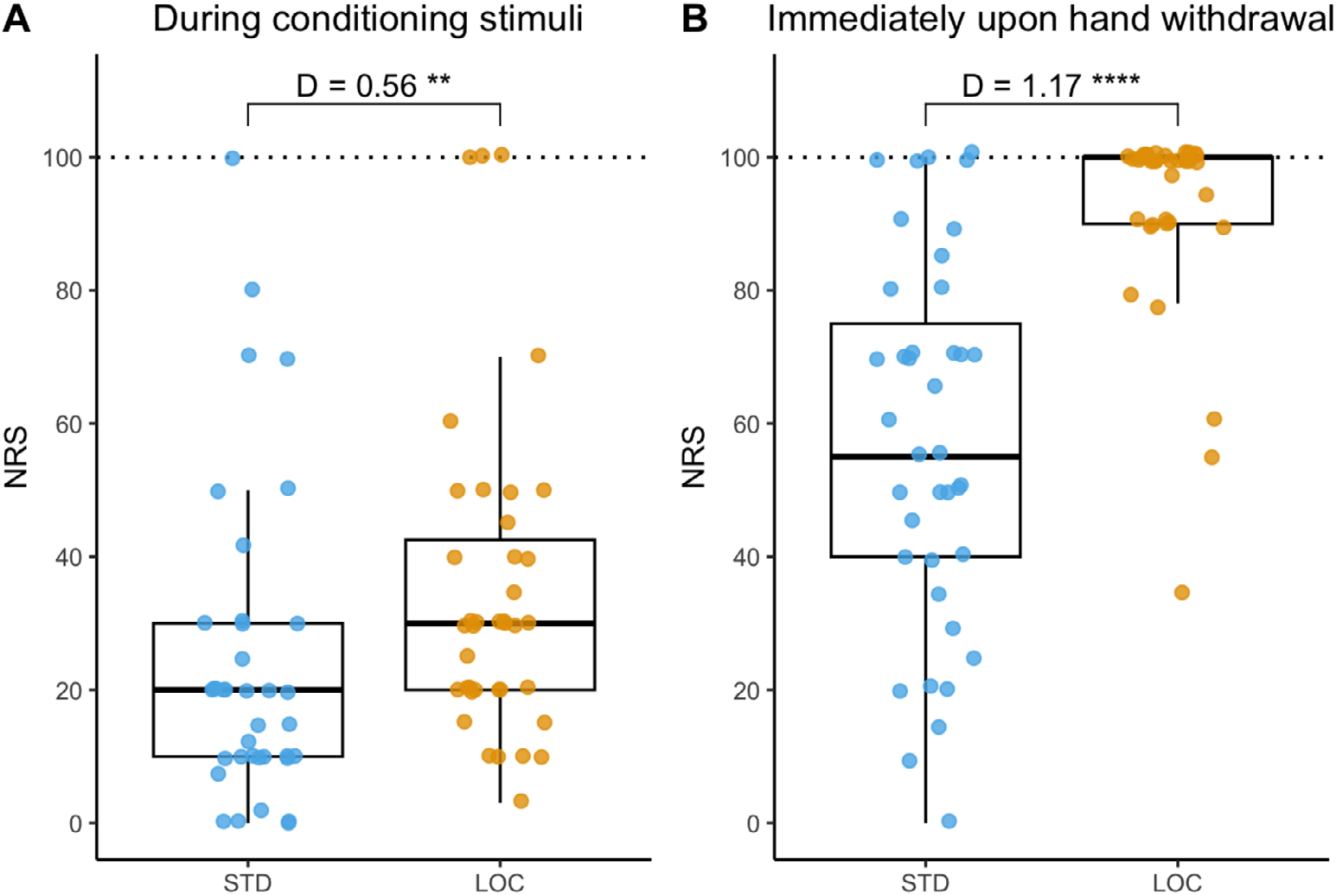
Pain rating for conditioning stimuli. Pain rating was obtained during conditioning stimuli (A) and immediately upon hand withdrawal (B). NRS: The numeric rating scale (0–100). LOC, local CPM test protocol (ice water conditioning); STD, standard CPM test protocol (7°C water conditioning). **p < 0.01, ****p < 0.0001.

The conditioning stimuli in the two CPM protocols differed in several characteristics. In STD (7°C water immersion), test stimuli started at 10 s; at this point, the pain rating of the conditioning stimuli was 23 ± 23.6 NRS (Figure 3A and Table 2). Subjects were instructed to withdraw their hands immediately after the mid‐point test stimuli (87.7 ± 20.6 s after immersion onset); at this point, 5/40 subjects (13%) had already reached tolerance (NRS = 100), the pain rating was 57.4 ± 27.8 NRS (Figure 3B and Table 2).

**TABLE 2:** Mean and variance of CPM outcomes and pain ratings of conditioning stimuli.

	STD (*n* = 39)	LOC (*n* = 39)	STD vs. LOC
	Female/Male [Cohen's *d*]	Female/Male [Cohen's *d*]	*p* (Cohen's *d*)
HPS_Pre_	All: 39.8 ± 16.7 41.0 ± 16.5/38.5 ± 17.3 [0.15]	n.a.	n.a.
HPS_Mid_	All: 31.5 ± 20.6 33.8 ± 22.6/29.1 ± 18.6 [0.23]	n.a.	n.a.
HPS_Post_	All: 34.5 ± 18.3 37.4 ± 20.0/31.4 ± 16.2 [0.33]	n.a.	n.a.
PPT_Pre_	All: 388 ± 157 370.5 ± 135.2/406.0 ± 179.6 [0.22]	All: 467 ± 126 492.5 ± 150.9/440.6 ± 90.5 [0.41]	0.004 (0.49)
PPT_Mid_	All: 452 ± 167 406.2 ± 141.0/500.8 ± 181.3 [0.58]	n.a.	n.a.
PPT_Post_	All: 410 ± 145 367.4 ± 104.7/455.6 ± 169.6 [0.63]	All: 557 ± 154 568.0 ± 171.4/545.2 ± 137.4 [0.15]	< 0.0001 (0.85)
NRS (at 10s during conditioning stimuli)	All: 23.0 ± 23.6 29.6 ± 23.3/16.2 ± 22.5 [0.58]	All: 34.8 ± 24.1 35.4 ± 22.0/34.2 ± 26.7 [0.05]	0.001 (0.56)
NRS (immediately upon hand withdraw)	All: 57.4 ± 27.8 50.0 ± 25.1/65.3 ± 28.9 [0.56]	All: 93.1 ± 14.1 96.9 ± 5.7/89.1 ± 18.9 [0.57]	< 0.0001 (1.17)
Testing time (s)	All: 87.7 ± 20.6 85.4 ± 10.7/90.1 ± 27.2 [0.23]	All: 95.0 ± 63.7 92.6 ± 62.0/97.5 ± 67.0 [0.08]	0.49 (0.11)

*Note:* Results are presented as mean ± SD. *p*‐values were calculated with *t*‐tests.

Abbreviations: HPS, heat pain sensitivity (suprathreshold rating); LOC, local CPM test protocol (ice water conditioning, testing at thenar muscle); NRS, pain rating of conditioning stimuli; PPT, pressure pain threshold; STD, standard CPM test protocol (7°C water conditioning, testing at trapezius muscle).

In LOC (ice water immersion) NRS at 10 s was already 34.8 ± 24.1 (*p* = 0.001 vs. 7°C cold water; Figure 3A and Table 2) and immersion continued until on average 95.0 ± 62.8 s (*p* = 0.49 vs. 7°C cold water); at this point, pain rating of the conditioning stimulus was 93.1 ± 14.1 NRS (*p* < 0.0001 vs. 7°C cold water; Figure 3B and Table 2). During ice water immersion, 24/39 subjects (62%) reached tolerance before the 180 s cutoff and 25/39 subjects (64%) reached tolerance at the time when the cold water exposure had terminated (*x* ^2^ = 19.55, *p* < 0.001 vs. cold water).

### 3.2 Effects of the Two Conditioning Stimuli on the Two Outcomes

**FIGURE 4:**
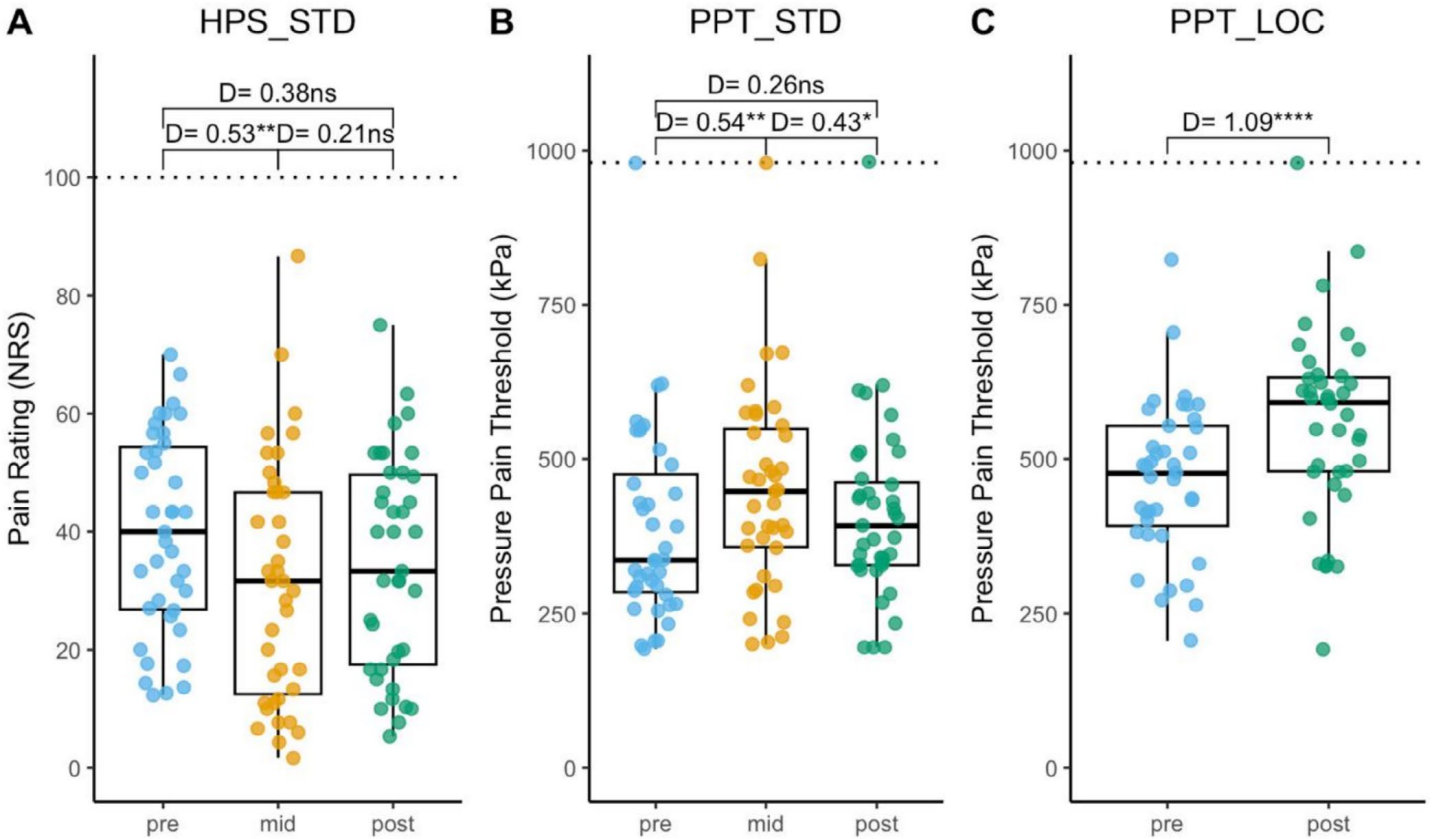
Changes in Pain Sensitivity across two CPM protocols. (A) Heat pain ratings in standard protocol (*N* = 39), (B) pressure pain thresholds in standard protocol (*N* = 39), (C) pressure pain thresholds in local test protocol (*N* = 39). CPM, conditioned pain modulation; HPS, Heat pain sensitivity (suprathreshold rating); kPa, Algometer pressure (range 0–980.7); LOC, local CPM test protocol (ice water conditioning); NRS, Numerical Rating Scale (range 0–100); STD, standard CPM test protocol (7°C water conditioning); PPT, pressure pain threshold. Dotted lines mark the upper limit of the assessment values. n.s.: No significant difference. D: Cohen's *d*. * *p* < 0.05, **p < 0.01, ****p < 0.0001.

Test stimuli in the CPM protocols were administered during the 10th second of water immersion (STD), and right after the hand was removed from the water (STD, LOC). Figure 4 illustrates the changes in HPS and PPT in both protocols (STD, LOC).

During conditioning 7°C cold water immersion, HPS was significantly reduced with moderate effect size (baseline to midterm: −21.8%; *D* = 0.53, *p* < 0.01), suggesting a meaningful suppression in perceived pain intensity (Figure 4A). After the hand withdrawal from the water bath, reduction of HPS was already slightly waning and, although still lower, did not differ significantly from baseline anymore (baseline to post: −11.1%; *D* = 0.38, *p* = 0.07). Likewise, PPT tested during immersion exhibited a significant increase with moderate effect size (baseline to midterm: +21.0%; *D* = 0.54, *p* < 0.01) and had also already partially recovered when assessed after withdrawal (midterm to post: *D* = 0.43, *p* < 0.05) and, although still higher, did not differ anymore significantly from baseline (baseline to post: +9.5%; *D* = 0.26, *p* = 0.57) (Figure 4B).

In contrast, in the LOC after 0°C ice water immersion, PPT increase was stronger with a very large effect size (baseline to post: +20.5%; *D* = 1.09, *p* < 0.001, Figure 4C). This was also highly significantly stronger than the threshold increase encountered after 7°C cold water exposure (+20.5% vs. +9.5%, *p* = 0.03).

### 3.3 Quantification of CPM Effect Sizes

**FIGURE 5:**
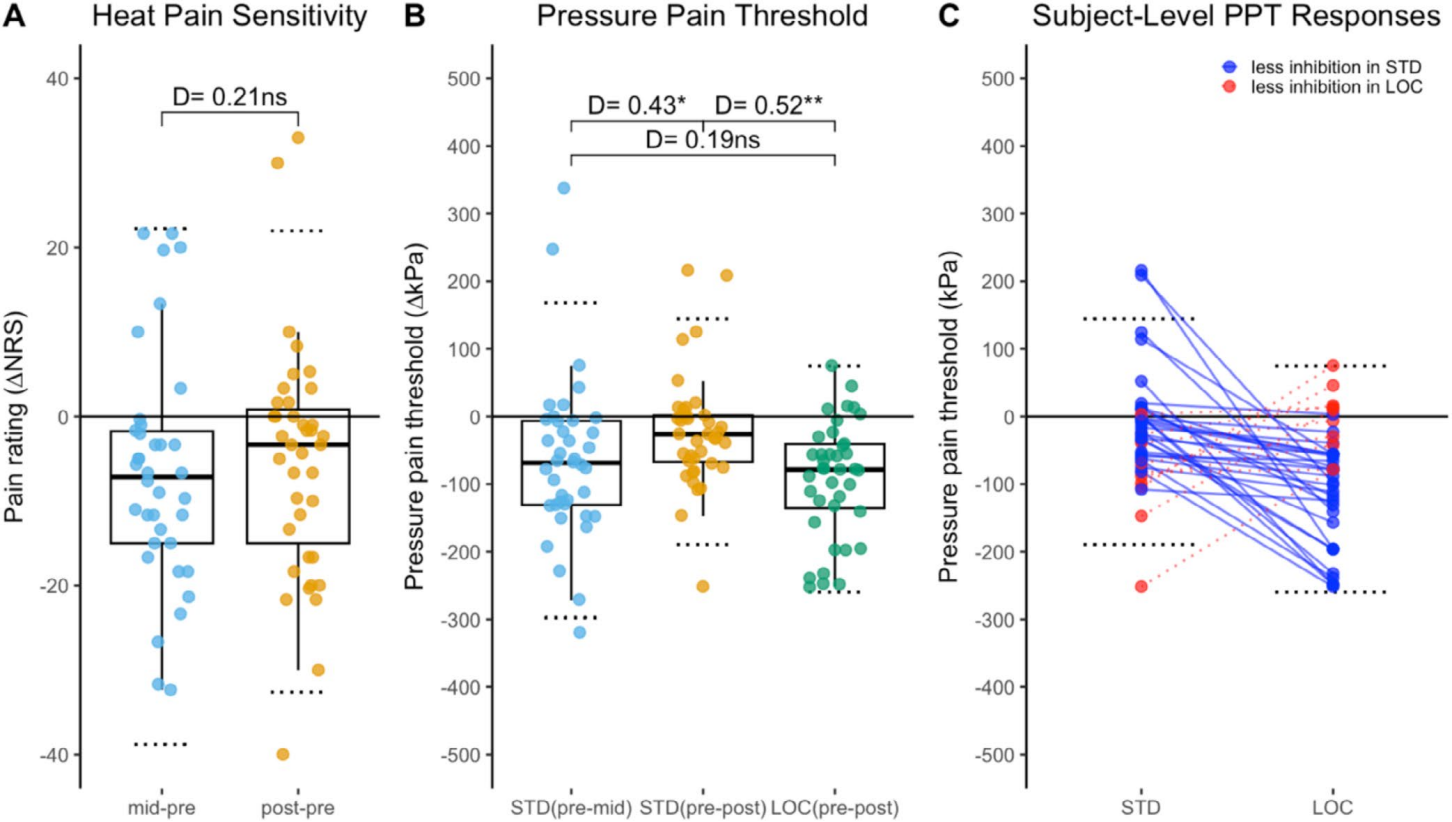
Conditioned pain modulation (CPM) effects. CPM effects were calculated as differences, such that negative values indicate inhibition. (A) Changes in heat pain sensitivity (HPS) in STD. (B) Changes in pressure pain threshold (PPT) in STD and LOC. (C) Comparison of CPM effects per subject for changes in PPT in STD vs. LOC. LOC, local CPM test protocol (ice water conditioning). CPM, conditioned pain modulation; STD, standard CPM test protocol (7°C water conditioning). D: Effect sizes as Cohen's *d* for paired comparisons (0.2 < small < 0.5 < medium < 0.8 < large), Bonferroni post hoc analysis. Dotted lines mark the upper and lower bounds of the 95% CI, representing the reference data of healthy subjects. *p < 0.05, **p < 0.01.

CPM effects were calculated as differences between pre and mid (STD) and pre and post (STD, LOC) in such a way that negative values indicate inhibition. Decreases in HPS in Figure 4 are hence depicted as negative values (Figure 5A). The CPM effect for HPS was smaller for post than mid (*D* = −0.21), but this difference was not significant. Increases in PPT in Figure 4 are depicted as negative values (Figure 5B). The CPM effect for STD was significantly smaller when assessed post vs. mid timepoint (*D* = −0.43, *p* < 0.05). LOC demonstrated a stronger CPM effect after conditioning than the STD (*D* = 0.52, *p* < 0.01).

In Figure 5C, individual changes in PPT (post vs. pre immersion) under STD and LOC protocols are compared by connecting lines illustrating pairs of PPT changes per subject. The majority of trials (*n* = 31) demonstrated stronger inhibitory effects in the LOC condition as for the group means, while in 8 trials the STD protocol induced a stronger inhibition condition.

### 3.4 Responder Rates and CPM Effect Reference Data

An intuitive way of calculating responder rates would be by counting the trials showing negative CPM effects in Figure 5, which means those showed analgesic effects. Accordingly, during midterm conditioning in STD, 32/39 subjects (82.1%) would be identified as responders, while the responder rate would drop to 26/39 (66.7%) after conditioning. PPT as an outcome measure in the same trials would return the same responder rate at midterm (32/39 = 82.1%) and a drop to 27/39 (69.2%) at the post conditioning assessment. The CPM effect of PPT in LOC would show only marginally higher responders (33/39 = 84.6%). The post‐conditioning responder rates would only differ by trend between STD and LOC (27/39 vs. 33/39; *x* ^2^ = 2.60, *p* = 0.11). While these raw responder rates were sufficiently high to yield mean negative CPM effect values on average, there was a certain number of subjects that exhibited facilitation instead of inhibition (dots with positive differences in Figure 5). Thus, facilitation per se does not indicate an abnormal outcome.

In order to derive a more reasonable range of normal variability of the CPM effects in both protocols for future use as reference data, we calculated the 95% confidence intervals as mean ± 1.96 × SD (dotted lines in Figure 5, numerical values in Table 3). This way, we propose that increases of HPS by more than 22.2/100 (mid‐pre) or 22.0/100 (post‐pre) are beyond the range of normal variability in young healthy adults and may hence indicate an abnormal outcome (loss of descending inhibition). Likewise, we propose that decreases of trapezius muscle PPT by more than 168 kPa (STD mid‐pre, Figure 5B) are beyond the range of normal variability in young healthy adults and may hence indicate abnormal loss of descending inhibition. For the LOC protocol, we propose that decreases of thenar muscles PPT by more than 75 kPa (LOC, post‐pre, Figure 5B) are beyond the range of normal variability in young healthy adults and may hence indicate an abnormal loss of descending inhibition.

**TABLE 3:** Variability of CPM effects in healthy subjects (95% confidence intervals).

	Mean Pre	Technically possible range	CPM effect timing	CPM effect Mean	CPM effect SD	Lower 95% CI cutoff	Upper 95% CI cutoff	Max facilitation technically possible	Max facilitation realistically possible
HPS_STD (NRS)	39.8	0–100	Mid‐pre	−8.3	15.6	−38.8	+22.2	+100	+60
			Post‐pre	−5.3	13.9	−32.6	+22.0	+100	+60
PPT_STD (kPa)	387.8	0–980.7	Pre‐mid	−64.5	118.8	−297.3	+168.4	+980.7	+387
			Pre‐post	−22.5	85.2	−189.6	+144.5	+980.7	+387
PPT_LOC (kPa)	467.2	0–980.7	Pre‐post	−92.0	85.4	−259.3	+75.3	+980.7	+467

*Note:* Range of CPM effects in healthy subjects was used to derive 95% confidence intervals (mean ± 1.96 × SD) to judge if individual patients are outside this reference range (‘abnormal CPM finding’). CPM effect timing: sign of differences was adjusted such that negative values indicate inhibition. Lower 95% CI Cutoff: maximum amount of inhibition in reference data. Upper 95% CI Cutoff: maximum amount of facilitation in reference data. Max facilitation technically possible: change across the full technically possible data range. Max facilitation realistically possible: change from mean pre value to max technically possible.

Abbreviations: HPS, heat pain sensitivity (suprathreshold rating); LOC, local CPM test protocol (ice water conditioning, one outcome: PPT, one time point: post); NRS, 0–100 numerical rating scale; PPT, pressure pain threshold; STD, standard CPM test protocol (7°C water conditioning, two outcomes: HPS and PPT, two time points: mid and post).

Clinical utility of such reference data depends on whether or not it is technically possible to make observations in patients that are outside the reference data range. The maximum possible increase in HPS would be from zero to 100 (i.e., 100), or more realistically from the mean baseline value of 39.8 to 100 (i.e., about 60, Table 3). Thus, in individual subjects increases in HPS ratings between the cutoff of the 95% CI (NRS increase by 22) and the maximum realistically expectable change (NRS increase by 60) would be interpretable as abnormal finding in the STD protocol.

The maximum possible decrease in PPT would be from 980 to zero kPa (i.e., 980 kPa) or more realistically from the mean baseline values of 387.8 kPa ( *M. trapezius* in STD) to zero. Thus, in individual subjects, decreases in PPT between the cutoff of the 95% CI (PPT decrease by 168 kPa) and the maximum realistically expectable change (PPT decrease by 387 kPa) would be interpretable as abnormal finding in the STD protocol (Table 3).

To illustrate the different potential sensitivities of the protocols, outcomes and timings for identifying abnormal findings in individual subjects, we also calculated CPM effects as relative differences (% changes from baseline). These adjust for differences in baseline values that might also exist between patient populations. Here we found that increases in HPS by more than 55% or decreases in PPT by more than 46% would be outside the 95% CI in the STD protocol. The best potential sensitivity to demonstrate loss of inhibition was by PPT in LOC, where decreases by more than 14% may be defined as abnormally facilitating. Note that this is more than the 5.3% decrease previously proposed to indicate any reliable CPM facilitation (in both healthy subjects and patients).

### 3.5 Test–Retest Reliability of CPM Effects

The test–retest reliability of CPM effects for HPS demonstrated moderate reliability of 0.77 (95% CI: 0.41–0.91) for the CPM effect assessed during conditioning. However, the HPS showed poor reliability for the CPM after‐effect, with values of 0 (95% CI: −1.53 to 0.60). In contrast, the PPT results in the STD and LOC exhibited good reliability both when tested during or after conditioning (Table 4).

**TABLE 4:** Reliability between visits in CPM effects and pain rating of conditioning stimuli.

		Visit 1	Visit 2	ICC (95% interval)
STD	HPS_Mid_‐HPS_Pre_	−6.1 ± 16.0	−10.5 ± 15.1	0.77 (0.41, 0.91)
	HPS_Post_‐HPS_Pre_	−0.8 ± 14.0	−10.1 ± 12.5	0.00 (−1.53, 0.60)
	PPT_Pre_‐PPT_Mid_	−0.06 ± 0.11	−0.08 ± 0.13	0.73 (0.32, 0.89)
	PPT_Pre_‐PPT_Post_	−0.01 ± 0.08	−0.05 ± 0.09	0.63 (0.06, 0.85)
	NRS 10s	27.6 ± 24.4	26.8 ± 22.8	0.47 (−0.34, 0.79)
	NRS post	59.2 ± 25.5	58.6 ± 28.3	0.71 (0.26, 0.88)
LOC	PPT_Pre_‐PPT_Post_	−0.08 ± 0.08	−0.07 ± 0.07	0.55 (−0.13, 0.82)
	NRS 10s	34.2 ± 22.6	35.4 ± 26.2	0.89 (0.72, 0.96)
	NRS post	93.0 ± 15.0	93.2 ± 13.7	0.83 (0.57, 0.93)

*Note:* Results are presented as mean ± SD.

Abbreviations: HPS, heat pain sensitivity (suprathreshold rating); LOC, local CPM test protocol (ice water conditioning); NRS, pain rating of conditioning stimuli; PPT, pressure pain threshold; STD, standard CPM test protocol (7°C water conditioning).

Test–retest reliability of the perceived painfulness of the conditioning stimuli is also shown in Table 4. It was higher in the LOC protocol (0°C ice water) than in the STD protocol (7°C water) at both 10 s and after hand withdrawal (*p* = 0.001; *p* < 0.0001, respectively).

### 3.6 Correlation Between CPM and Psychological Data

**FIGURE 6:**
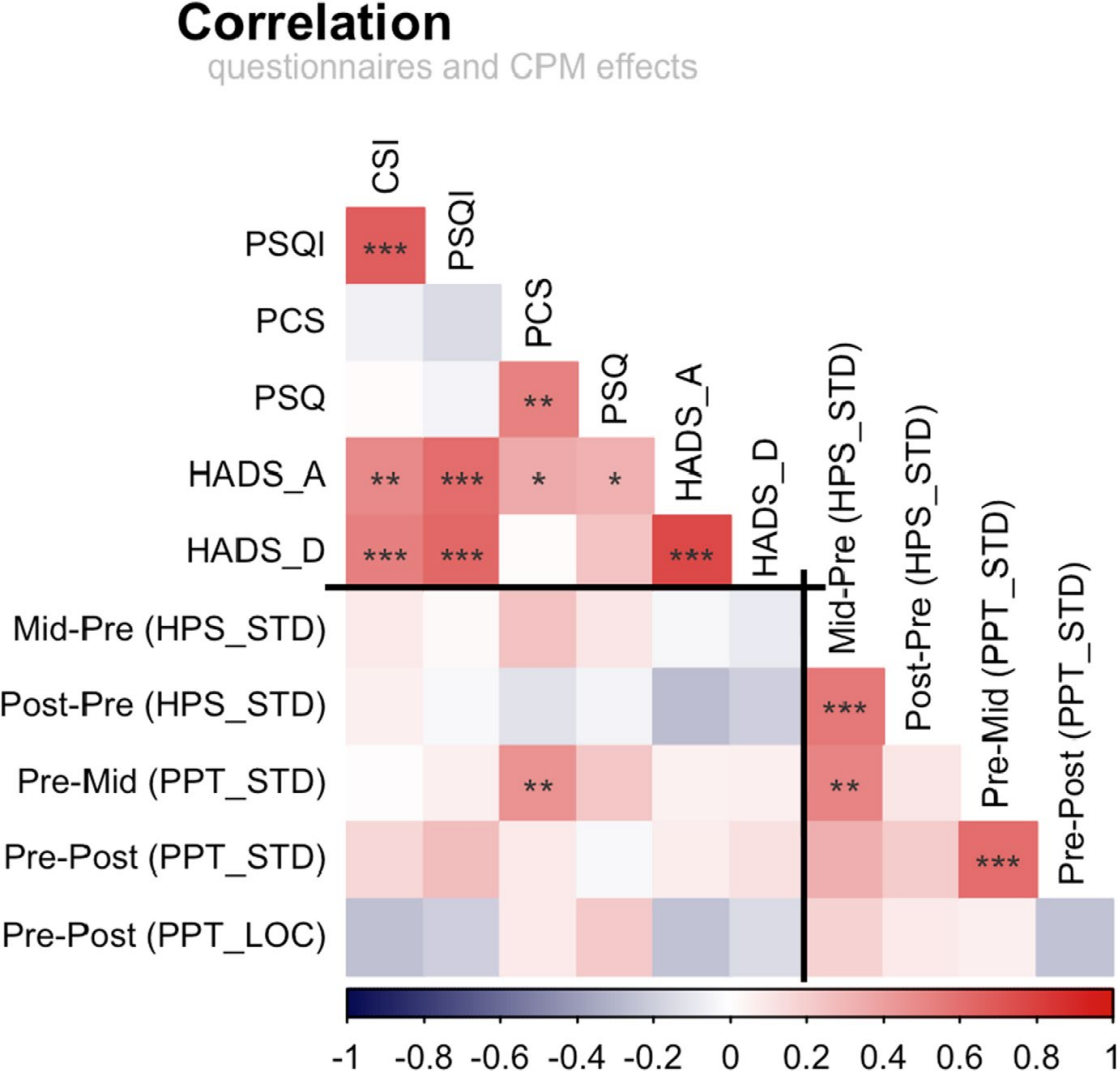
Correlation between questionnaires and CPM effects (*n* = 39). The relationship between the variables was analysed using the Pearson correlation coefficient. CSI, Central Sensitization Inventory; HADS_A, Hospital Anxiety and Depression Scale, anxiety score; HADS_D, Hospital Anxiety and Depression Scale, depression score; HPS, heat pain sensitivity; LOC, local CPM test protocol; PCS, Pain Catastrophizing Scale; PPT, pressure pain threshold; PSQ, Pain Sensitivity Questionnaire; PSQI, Pittsburgh Sleep Quality Index; STD, standard test protocol. *p < 0.05, **p < 0.01, ***p < 0.001.

Since there is some clinical evidence for a relationship between psychological predictors and CPM efficacy, we performed an exploratory correlation analysis (Figure 6). Within the CPM domain of the STD protocol (Figure 6, bottom right), there were positive correlations between the different outcomes. HPS during and after conditioning was highly correlated (*r* = 0.562, *p* < 0.001). Likewise, PPT during and after conditioning was correlated (*r* = 0.616, *p* < 0.001). A correlation of HPS with PPT was only found for the midterm (*r* = 0.515, *p* = 0.002). Across protocols, only PPT after conditioning could be compared in a meaningful way. The lack of correlation here probably reflects the lack of efficacy of STD at this time point (see Figure 4B) and hence systematic variance in LOC was compared only with error variance in STD.

Across psychological and CPM domains (Figure 6, bottom left), the Pain Catastrophizing Scale correlated with a CPM effect outcome, but only for PPT assessed during 7°C water immersion (*r* = 0.455, *p* = 0.004). This was, however, not a stable relationship because this correlation was almost completely absent in the 0°C ice water immersion, indicating that it may be related to divided attention rather than descending inhibition.

## 4 Discussion

The main findings of this study were: Conditioning with ice water (LOC) led to stronger inhibition of pressure pain threshold (PPT) than conditioning with 7°C water (STD) when tested immediately after conditioning. When tested during conditioning, effects of 7°C water immersion on heat pain sensitivity (HPS) had similar magnitude and test–retest reliability as those on PPT. For all outcomes assessed, 95% confidence intervals of the range of CPM effects in healthy subjects included a certain degree of facilitation instead of inhibition. PPT assessed after conditioning with ice water exhibited the narrowest confidence interval and cutoff for abnormal facilitation (decrease by more than 75 kPa or 14% of baseline PPT). Among paradigms tested, this was potentially the most sensitive for detecting abnormal loss of CPM inhibition in patients. Among psychological predictors, pain catastrophizing correlated positively with facilitation in the parallel CPM protocol and with subjective reports of sensitivity to imagined painful situations.

### 4.1 CPM Responder Rates and Ranges of Normal Variability

At a group level, healthy subjects usually exhibit net inhibition in CPM paradigms, whereas net facilitation in patients at the group level has been interpreted as deficits in descending inhibitory controls (Fernandes et al. 2019; Gerhardt et al. 2017; Kennedy et al. 2020; Lewis et al. 2012; Schliessbach et al. 2023). This logic cannot be applied to individual subjects because, for all combinations of conditioning stimulus, CPM outcome, and timing, we and others have found that some healthy subjects facilitated rather than inhibited. Moreover, in many chronic pain conditions such as fibromyalgia, there is heterogeneity in the phenotypes of individual patients. It is therefore highly desirable to be able to use a robust CPM paradigm that permits conclusions on whether or not a particular patient's finding is within the range of normal variability (Vincenot et al. 2025).

For this purpose, we followed the footsteps of standardization of static Quantitative Sensory Testing (QST) by DFNS that used 95% confidence intervals in healthy subjects to establish reference data and make QST clinically applicable to individual subjects (Rolke, Magerl, et al. 2006). All 95% CI extended into the positive range (i.e., included some degree of facilitation), but we could delimit a maximum extent of facilitation that would still be within the range of normal variability of healthy subjects. Any facilitation beyond that value may indicate abnormal CPM inhibition loss in individual patients. Because baseline values differ according to test site and patient population, we recommend calculating reference data as % change. The smallest 95% CI was found for PPT in LOC, that is, after ice water. Here about a 14% decrease in threshold would be considered an abnormal test result. Using this cutoff, a retrospective re‐analysis of a previous clinical study on the effects of spinal cord stimulation on CPM (Schuh‐Hofer, Eichhorn, et al. 2018) indicates that these chronic pain patients had individually abnormal CPM when their spinal cord stimulator was off, but moved into the range of reference data when the stimulator was on. Another study in chronic knee pain similarly showed that CPM facilitation and inhibition can occur within individuals, highlighting their relevance as mechanistic markers for precision‐based interventions (Larsen et al. 2024). It should be emphasized that a single abnormal test result does not prove pathology, since by definition 5% of healthy people will show such results, but it provides diagnostic mechanistic hints on functional changes in descending pain modulation.

### 4.2 Choice of Conditioning and Test Stimuli

The aftereffects of conditioning ice water on PPT were significantly higher than those of 7°C cold water. Given that the painfulness of the conditioning stimulus was also higher for ice water (93/100) than for 7°C cold water (57/100), our data are consistent with previous studies that reported a positive relation between conditioning pain intensity and CPM effect (Graven‐Nielsen et al. 2017; Nir et al. 2010); other studies found that conditioning stimuli need to be painful to induce net inhibition, but did not find a direct proportionality for conditioning pain and CPM effect (Granot et al. 2008; Martel et al. 2024). Test–retest reliability of the painfulness of the conditioning stimulus was also higher for ice water. But this did not lead to a higher reliability of the CPM effect assessed by PPT immediately after conditioning. Thus, there may be a trade‐off between sensitivity to change and test–retest reliability.

PPT demonstrated consistent reliability across both STD and LOC conditions, highlighting its utility as a measure for evaluating pain modulation. When used as part of the QST protocol of the DFNS, PPT assessed over thenar muscles has a low natural variability across subjects (figure 1 in Rolke, Magerl, et al. 2006), excellent test–retest reliability (*r* = 0.881; Geber et al. 2011), and high clinical utility due to its sensitivity to change in various diseases (Maier et al. 2010). The significant increase in PPT observed at midterm under the STD condition and at posttest under the LOC condition underscores the sensitivity of PPT measurements in detecting changes in evoked pain sensitivity over time. This finding aligns with existing literature suggesting PPT as a reliable indicator of alterations in nociceptive processing (Bilika et al. 2024; Kovacevic et al. 2021). PPT assessment over thenar muscles is more easily performed and yields less variable results than trapezius; since CPM effects are systemic, thenar is a useful test site for improved reliability and interpretability outside a clinically affected region.

During conditioning, HPS had similar sensitivity to change and reliability as PPT, but effects were short‐lived and undetectable after conditioning. As a caveat, we have to mention that HPS was always tested after PPT, so aftereffects of HPS might be detectable immediately after conditioning. Actual ratings for HPS differed from 50/100 on NRS when the pre‐calibrated test stimulus was given at a later point in time (at baseline). This underlines that standardization of suprathreshold test stimuli is not simple. Threshold determination avoids this problem and may hence be preferable for practical purposes.

### 4.3 Timing of Assessing the CPM Effect

DNIC is known to induce only short‐lasting aftereffects for a few minutes (Schuh‐Hofer, Fischer, et al. 2018; Villanueva and Le Bars 1995). To maximize effect sizes, many authors choose to assess CPM efficacy while the conditioning pain is still ongoing. In fact, in our data, the CPM effect was pronounced during the test phase but diminished rapidly after the withdrawal from the painful stimulus in the STD condition. PPT assessed at mid‐term also had better test–retest reliability than when assessed after the end of the conditioning stimulus. CPM assessment during conditioning stimulation has thus been preferred by some authors (Granovsky et al. 2015), while other studies suggest that sequential protocols may yield stronger CPM effects (Billens et al. 2025).

For a mechanistic interpretation, simultaneous CPM paradigms have a major disadvantage: they are confounded by divided attention mechanisms involving thalamo‐cortical and spinal processing. Simultaneous CPM paradigms cannot be interpreted as a specific test of descending brainstem controls (Plaghki et al. 1994; Reinert et al. 2000; Sprenger et al. 2012; Torta et al. 2019). Our findings support this notion, as the CPM effect on PPT after conditioning in LOC vs. during conditioning in STD showed no correlation. Moreover, catastrophizing only modulated CPM effects in the simultaneous STD protocol, presumably via attentional control. We recommend a sufficiently strong conditioning stimulus to obtain significant aftereffects and rapid post‐conditioning assessment.

### 4.4 Factors Contributing to the CPM Effect Variance

Several subject‐related factors can influence CPM outcomes, including age, sex, race, and hormonal variations (Eichhorn et al. 2018; Ibancos‐Losada et al. 2020; Nuwailati et al. 2022; Riley et al. 2020). While some studies have reported that younger adults tend to exhibit stronger CPM effects (Nuwailati et al. 2022), others have found only weak association between demographic factors and CPM responses (Ibancos‐Losada et al. 2020; Riley et al. 2020). For CPM reference data, stratification by age and sex should be attempted in a similar way as for static QST (Magerl et al. 2010).

The positive correlation between the Pain Catastrophizing Scale (PCS) and CPM effect in the STD protocol suggests that individuals with higher pain catastrophizing tendencies may experience less descending inhibition. However, this relationship was only present when CPM effect was assessed during the conditioning stimulus in the STD protocol. It may thus be driven by heightened attention to pain, where individuals with higher PCS scores are more engaged with the test stimuli. Individuals who report higher levels of pain catastrophizing demonstrate diminished endogenous pain inhibition: higher catastrophizing scores negatively correlated with endogenous pain inhibition magnitude (Goodin et al. 2009; Weissman‐Fogel et al. 2008). Since pain catastrophizing occurs at the brain level, it may exert its influence through cortical regions, which play a key role in attentional control. These thalamo‐cortical and cortico‐cortical mechanisms may interact with top‐down pain modulation via brainstem descending inhibitory pathways. In fact, we had previously observed that healthy students with high catastrophizing scores had better recall of previously experienced ice‐water pain (Pallegama et al. 2017).

PCS may serve as an indirect marker of attentional engagement with pain modulation mechanisms, highlighting the potential influence of psychological factors on endogenous pain control processes, but these processes are likely distinct from descending brainstem controls.

### 4.5 Limitations and Further Studies

As a limitation, our sample was small and too homogenous to permit calculation of stratified reference data for age, sex or other factors. Such stratification should be done in future multi‐centre studies (cf. Magerl et al. 2010). We successfully implemented a standard laboratory medicine approach to CPM, serving as a model for larger studies. From a clinical perspective, pressure pain threshold modulation at the thenar by contralateral ice water immersion showed the highest potential sensitivity to demonstrate abnormal CPM findings at an individual level, which could improve pain diagnostics and treatment monitoring. The painfulness of this conditioning stimulus might limit its application in chronic pain populations. However, conditioning stimulus duration is adjusted to individual pain tolerance (180 s cutoff for insensitive subjects; Martel et al. 2024); this allowed application in a clinical population (Schuh‐Hofer, Eichhorn, et al. 2018).

## 5 Conclusion

Our study demonstrates that the standard test protocol (STD) proposed by Granovsky et al. 2022 yielded medium to large effect sizes and good test–retest reliability when mechanical or heat pain sensitivity were tested simultaneously with the mild cooling conditioning stimulus (7°C water immersion) (Granovsky et al. 2022). These effects, however were only transient and not significant when tested immediately after the end of the conditioning stimuli. Thus their interpretation remains limited due to confounding effects of divided attention. We support the use of a somewhat stronger conditioning stimulus (ice water immersion) with exposure time shorter than or up to individual tolerance levels. Pressure pain threshold (PPT) proved to be a more reliable and effective measure of pain modulation compared to heat pain sensitivity (HPS), with higher test–retest reliability and stronger effect sizes. This may be related to its excellent reliability in particular when assessed over thenar muscles.

For future clinical use of CPM in evaluating individual patients, we piloted the calculation of 95% confidence intervals in healthy subjects. Such intervals include a certain degree of facilitation, but the CPM paradigm is technically able to document facilitation beyond those cutoffs, indicating its potential to transition CPM assessments from group‐level research to individualized clinical practice. Future studies should validate these findings through multicentre trials and investigations incorporating age‐ and sex‐adjusted reference ranges to enhance the clinical applicability of CPM assessments.

## Author Contributions

**Yi‐Wun Lin:** conceptualization, methodology, software, validation, formal analysis, investigation, data curation, writing – original draft, visualization, project administration, funding acquisition. **Hannah Schmidt:** software, formal analysis. **Niko Möller‐Grell:** investigation. **Walter Magerl:** methodology, review and editing. **Li‐Ling Hope Pan:** review and editing. **Shuu‐Jiun Wang:** review and editing. **Li‐Wei Chou:** conceptualization, writing – review and editing, supervision. **Rolf‐Detlef Treede:** conceptualization, methodology, validation, resources, writing – original draft, supervision, funding acquisition.

## Funding

This study was supported by DFG (SFB1158 project B09 and S01) and DAAD‐NSTC (113–2917‐I‐A49A‐002).

## Disclosure

Plagiarism Declaration: We hereby declare that this paper is our own work, except where acknowledged, and has not been submitted elsewhere.

## Conflicts of Interest

The authors declare no conflicts of interest.

## Data Availability

De‐identified data will be available upon reasonable request to the corresponding author.
